# The Impact of Immunotherapy on Sleep and Circadian Rhythms in Patients with Cancer

**DOI:** 10.3389/fonc.2023.1295267

**Published:** 2023-11-27

**Authors:** Diwakar D. Balachandran, Lara Bashoura, Ajay Sheshadri, Ellen Manzullo, Saadia A. Faiz

**Affiliations:** ^1^ Department of Pulmonary Medicine, The University of Texas MD Anderson Cancer Center, Houston, TX, United States; ^2^ Department of Internal Medicine, The University of Texas MD Anderson Cancer Center, Houston, TX, United States

**Keywords:** immunotherapy, sleep, circadian rhythm, immune checkpoint inhibitors, immunity

## Abstract

Immunotherapy has revolutionized treatments for both early and advanced cancers, and as their role evolves, their impact on sleep and circadian rhythms continues to unfold. The recognition, evaluation, and treatment of sleep and circadian rhythm disturbance leads to improved symptom management, quality of life and treatment outcomes. An intricate complex relationship exists in the microenvironment with immunity, sleep and the tumor, and these may further vary based on the cancer, addition of standard chemotherapy, and pre-existing patient factors. Sleep and circadian rhythms may offer tools to better utilize immunotherapy in the care of cancer patients, leading to better treatment outcome, reduced symptom burden, and increased quality of life.

## Introduction

**Figure 1:**
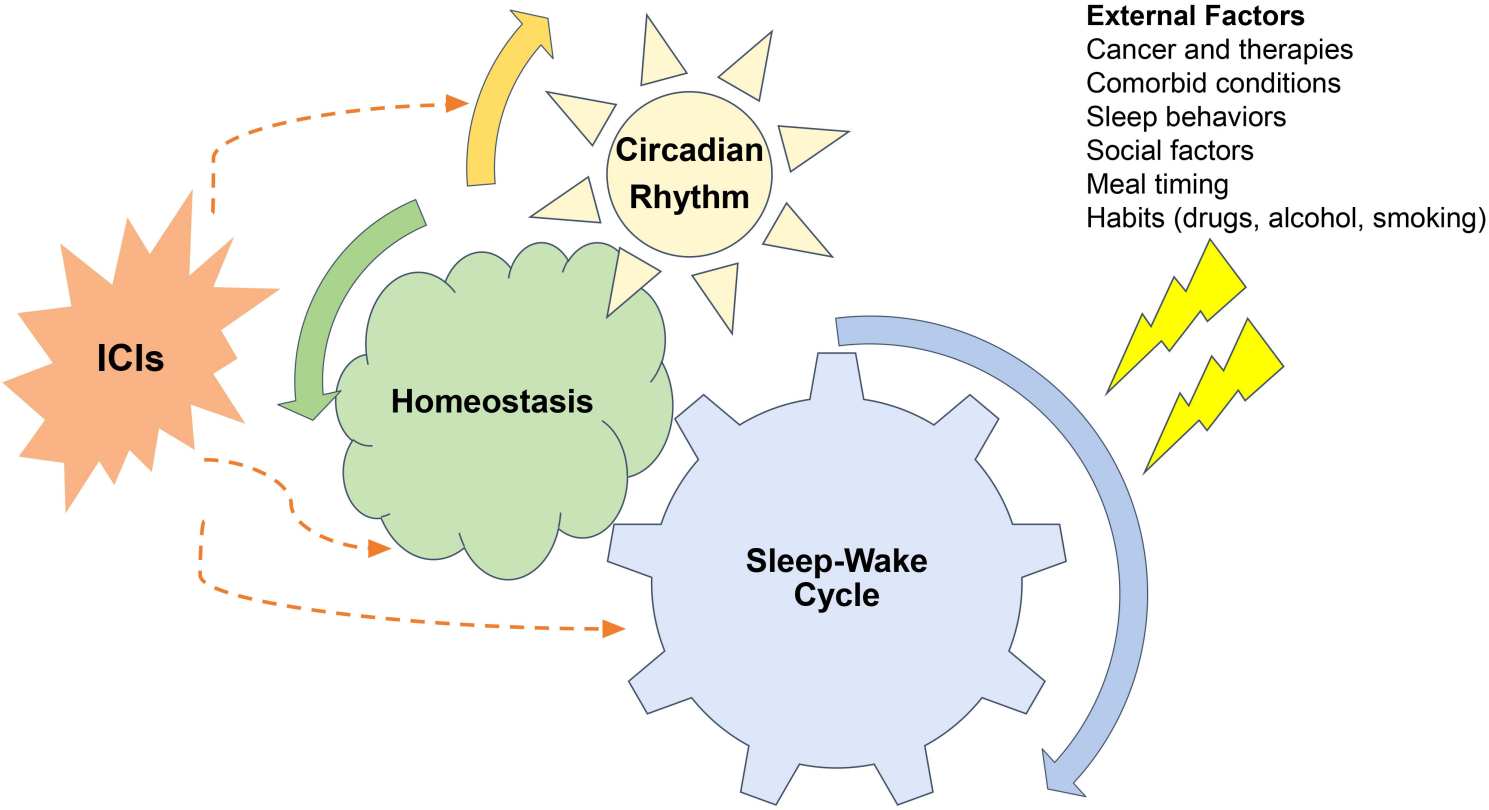
Relationships between sleep-wake cycles, circadian rhythms, cancer and immune checkpoint inhibitors (ICI). Sleep wake cycles are controlled by the brains neural networks and are governed by circadian rhythms. External factors related to cancer, comorbid conditions, sleep behaviors, social factors, meal timings, and other habits all can perturb both sleep-wake cycles and circadian rhythms which disturbing homeostasis. These disturbances impact the function of immune checkpoint inhibitors both on their on-target therapeutic and off- target auto-immune effects.

Immunotherapy has revolutionized treatments for both early and advanced cancers, and as their role evolves, their impact on sleep and circadian rhythms continues to unfold (1, 2). Sleep disturbance may occur at any time during the spectrum of the cancer care continuum including prior to diagnosis, during treatment and years into survivorship. Sleep disruption during cancer treatment can perpetuate and exacerbate the symptom burden for cancer patients (3–6). The recognition, evaluation, and treatment of sleep and circadian rhythm disturbance leads to improved symptom management, quality of life and treatment outcomes (7). Immune checkpoint inhibitors (ICIs) modulate the immune system to target cancer cells, and the interaction between immunity, sleep and circadian rhythm has been well described. Thus, ICIs likely contribute another dimension to the complex interactions between cancer and sleep (3, 4, 7–10). Our objectives are to review the importance of sleep and circadian rhythms in cancer care, to discuss the interplay between sleep, circadian rhythms and immunity and to highlight the interplay of ICIs with sleep, circadian rhythms, immunity and symptom burden (**Figure 1**).

## Immune checkpoint inhibitors

ICI’s main function is to disable T-cell regulation to amplify the impact of the T-cell mediated killing of cancer cells. Immune checkpoints are immune cell functions governed by receptor-ligand interactions which control the activation or inhibition of immune responses. Activation of the immune system is a desired outcome to achieve tumor control but can also lead to autoimmunity and toxicity (11). The discovery of monoclonal antibodies against the inhibitor immune checkpoint CTLA–4 (cytotoxic T lymphocyte antigen 4), PD-1 and PD-L11 (programmed cell death 1 and programmed cell death ligand-1, respectively) have resulted in dramatic antitumor responses by the upregulation of immune activation at various stages of the immune cycle (12).

ICI therapy has been transformational in the care of cancer patients, and they have become a pillar for cancer care including neoadjuvant, adjuvant, primary therapy and the treatment of metastatic disease of numerous cancer types (12–14). Now more than a decade after the Federal Drug Administration (FDA) approval of the first ICI, there is nascent recognition of their impact that these medications may have on sleep and circadian rhythms (15, 16). Since the approval of ipilimumab to treat metastatic melanoma in 2011, several ICIs have been approved to treat a growing list of cancers, and many regimens even include dual immunotherapy to maximize impact. The efficacy and safety have been established in a myriad of clinical trials, but immune-mediated adverse events (irAEs) resulting in organ dysfunction resembling autoimmune diseases can occur (17). The most common irAEs include rash, diarrhea and fatigue, but endocrinopathies, myocarditis, hepatitis, pneumonitis, nephritis and nervous system issues may also occur (18). Thus, ICIs, their irAEs and the underlying cancer may all impact sleep.

## Fundamentals of sleep and circadian rhythms

Sleep is a fundamental and basic need for life. Sleep is formally studied by polysomnography and is divided into both non-rapid eye movement (NREM) and rapid eye movement (REM) sleep (19). NREM sleep is further divided into 3 stages including: Stage 1, light sleep or drowsiness; Stage 2 which comprises the largest portion of non-REM sleep; Stage 3, deep or slow-wave sleep. The sensitivity of the cortical response to auditory, tactile, and visual stimuli are correspondingly more depressed during stage 3 sleep compared with stage 1 sleep. REM sleep or paradoxical sleep is a metabolically active period of sleep with saccadic rapid eye movements as the hallmark of this important stage of sleep. Both NREM and REM sleep have critical restorative functions and are essential for cognition, learning, and memory (20). Sleep impacts immune function in a fundamental manner affecting both innate and adaptive immunity (21). Immune function is enhanced by both better sleep quality and optimal sleep duration (20). Perturbations of sleep quality and duration fundamentally impact health and disease and have been shown to impact cancer incidence, cancer-related symptoms, and cancer outcomes (10). Identification of these of these symptoms and disorders is enhanced by the utilization of validated subjective and objective tools to assess sleep and circadian rhythms (**Table 1**).

**Table 1:** Tools to evaluate sleep and circadian rhythms.

Tool	Significance
**Patient-reported outcomes**	**Pittsburgh Sleep Quality Index Questionnaire.** A survey that measures sleep quality over the last 1 month. With a maximum score of 24, a score ≥ 5 represents disturbed sleep (22)
	**Epworth Sleepiness Scale.** An 8-question survey which measures daytime sleepiness. With a maximum score of 24, a score ≥ 10 represents increased daytime sleepiness (23).
	**Insomnia Severity Index.** A 7-question survey with maximum score of 28 with a score ≥15 indicative of moderate clinical insomnia (24).
	**STOP-BANG Questionnaire.** An 8-question tool incorporating symptoms (snoring, fatigue), medical history (hypertension), and anthropometric data (age, gender, BMI, neck circumference). With a maximum score of 8, total signifies the following: ■ a score of <3 low risk for OSA ■ ≥3 and <5 intermediate risk for OSA ■ ;≥5 high risk for OSA (25).
**Diagnostic testing**	Polysomnography: a multi-parameter sleep test recoding EEG, EOG, and EMG, respiratory and cardiac data to evaluate sleep and sleep disorders. Usually, facility based (26).
	Ambulatory sleep testing: multi-parameter sleep testing done at home, primarily to detect sleep apnea (27).
	Actigraphy: wrist-worn accelerometer which measure activity count and can be used to evaluate sleep parameters such as total sleep or rest time, sleep latency, sleep efficiency and circadian rhythms (28).

BMI, body mass index; OSA, obstructive sleep apnea.

**Figure 2:**
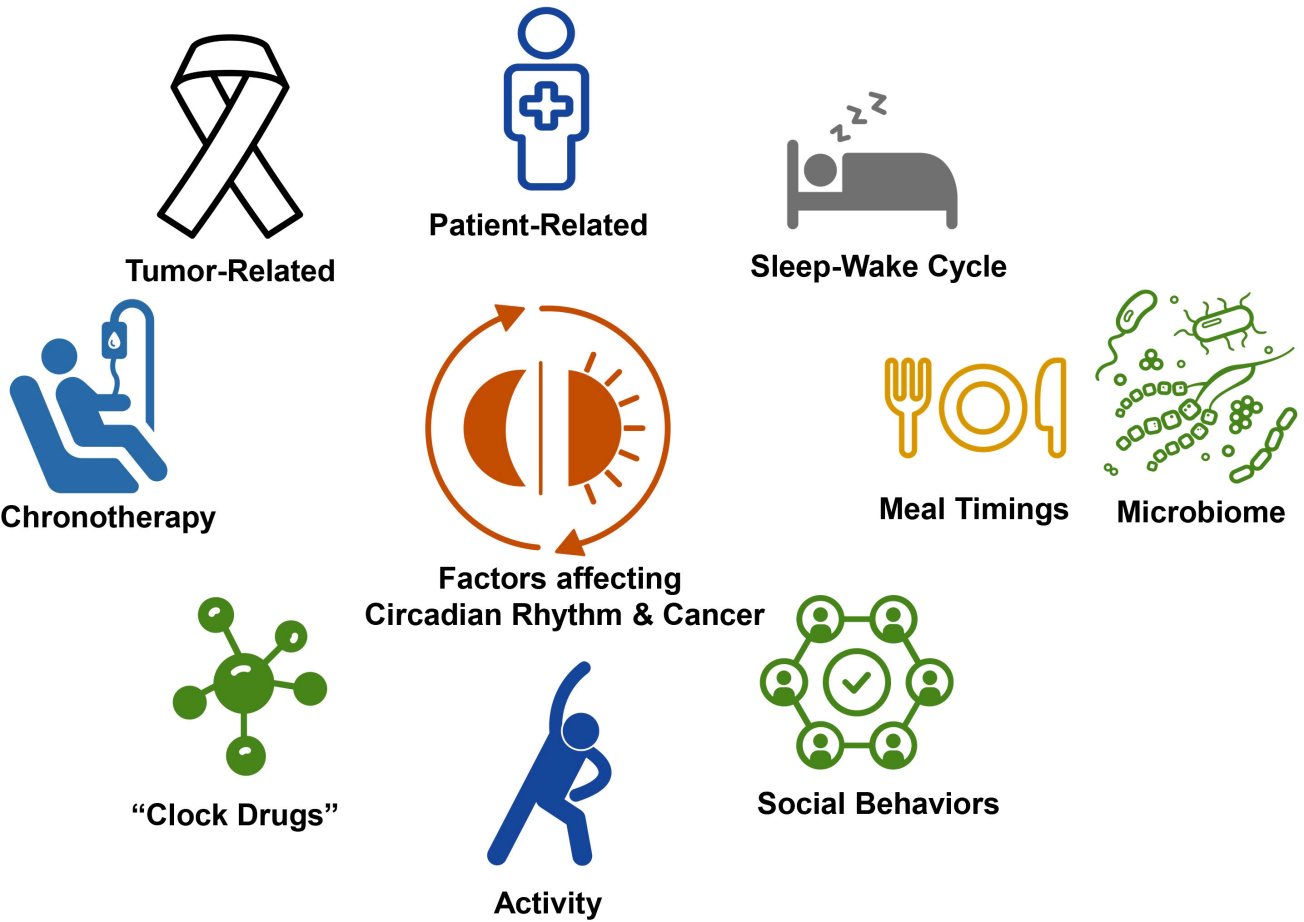
Factors impacting circadian rhythms and cancer. The intersection between circadian, rhythms, and cancer is governed by multiple factors, including patient related factors, such as comorbidities and habits and tumor, related factors. Activity, social behaviors, and meal timings entrain circadian rhythms and sleep wake cycles and may be targets for chronoregulation. There is increasing recognition that the microbiome is bi-directionally impacted by circadian rhythms and sleep-wake cycles. Further opportunity for intervention using circadian rhythm related strategies include chronotherapy and the development of clock drugs, the targeting clock gene expression and activity.

The control of sleep is determined by a defined set of hypothalamic and brainstem nuclei primarily with pathways to the thalamus and cortex and is best explained by the 2 process models of sleep regulation with both homeostatic and circadian control. Homeostatic control refers to the concept of “sleep debt” which accrues during wakefulness increases the impetus to sleep (29). Circadian, meaning about a day, rhythms are an approximately 24-hour cyclic rhythm which govern both sleep and wakefulness and are a critical input into sleep regulation and helps maintain and consolidate nocturnal sleep as the homeostatic process abates during the sleep period, as sleep debt is paid off (30). Circadian rhythms originate in the suprachiasmatic nucleus in the brain and fundamentally control biology on a behavioral, physiologic, cellular, and molecular level. Circadian rhythms also drive the visible behaviors of sleep and wakefulness (30). Disrupted circadian rhythms have also been shown to impact health and disease, including cancer incidence, cancer symptoms, and cancer outcomes (31). Furthermore, immune regulation and many aspects especially cellular immunity are also under circadian control (32). These relationships provide a basis on which to better understand the intersection between the circadian clock, cancer and immunotherapy (**Figure 2**).

## Sleep, circadian rhythms, and cancer

Sleep and circadian disruption are considered risk factors for cancer (33). Sleep disruption is at least 3 times as prevalent in cancer population as in the general population (34). Sleep and circadian disruption have also been shown increase with the severity of disease and the degree of disruption has prognostic value (31). Furthermore, sleep disruption clusters with other symptoms of cancer including pain, mood disturbance, and fatigue (9). Improved sleep quality in cancer patients is associated with a better prognosis and treatment response. Innominate and colleagues demonstrated an 8-month increase in overall survival in patients receiving chemotherapy for metastatic colon cancer who were shown to be objectively at rest by actigraphy during their time in bed (35). Sleep disorders such as obstructive sleep apnea has been shown to increase both cancer incidence and mortality (36, 37). Gozal posits that sleep fragmentation is oncogenic and hypothesizes several mechanisms for this relationship through sleep disruption’s impact on inflammation and immunity, the autonomic nervous system, the hypothalamic- pituitary axis, oxidative stress and hormonal pathways (33, 38).

The circadian clock is a major regulatory pathway for the cell cycle. Clock genes regulate the cell cycle and therefore modulate gene replication, gene expression, and cellular proliferation (39). In a chronic jet lag model, spontaneous hepatocellular carcinoma occurred in mice following a mechanism very similar to that observed in obese humans (40). Genetic abnormalities in clock genes have been shown to be both oncogenic as well as onco-suppressive (41–49). In studies with circadian clock gene mutant animal models, clock gene BMAL1 deficient knockout mice were shown to have greater progression of hematologic malignant disease and breast cancer cell metastasis (50, 51). Furthermore, core circadian genes Per2 and Bmal1 were shown to have cell-autonomous tumor-suppressive roles in transformation and lung tumor progression (52). Disrupted circadian rhythms in humans have consequences for health and disease and highlight the importance of maintaining robust circadian rhythms of sleep and wakefulness (20). Shift work has been classified as a carcinogen due the higher rate of cancer (53, 54). Circadian rhythm has also been shown play a role in the overall symptom burden of cancer and modify levels of fatigue, depression, and sleep disturbance. Circadian rhythm disturbance has been demonstrated as common regulating factor in the manifestation symptom clusters (35, 55–59). Cancer cells carry mutations which disassociate cells from normal circadian control of the cell cycle compared with heathy tissue, and this difference is used to amplify the therapeutic window in cancer chronotherapy (60, 61). Cancer chronotherapy has been shown to improve on-target effects while minimizing off-target adverse effects (31, 62, 63). Circadian rhythm disruption has prognostic value as cancer patients with attenuated circadian rhythms often also have higher mortality (64). These factors show that circadian rhythms are important not only for behavioral control of sleep but for the regulation and treatment of cancer (39).

The impact of sleep and circadian rhythms in cancer patients is primarily twofold. First, quality of life is often dramatically impaired due to insomnia, excessive daytime sleepiness, and fatigue. These often debilitating symptoms decrease opportunities for beneficial lifestyle habits such as exercise, social interaction, and healthy diet (65). Second, sleep and circadian rhythms are coupled to improved symptom control, better treatment outcomes, and extended survival in cancer patients. About 30 to 55% of cancer patients have impaired circadian rest-activity rhythms with attenuated rhythms correlating to more advanced and aggressive cancer. Interventions to improve both sleep disorders and circadian rhythm disorders have been shown to ameliorate cancer symptoms and may impact cancer outcomes (66–68).

Circadian rhythms regulate both immunity as well as the cycle of hormones such as cortisol which impacts immune cell function, and through this control of cortisol secretion, the circadian clock function as a gate that controls many aspects of immune function in cancer including cancer cell antigen release and presentation, activation of effector immune cells, trafficking, tumor infiltration and elimination of cancer cells. These regulatory rules in both tumor surveillance and prevention of oncogenesis highlight the circadian clock’s relevance to cancer immunotherapy (69).

## Sleep, circadian rhythms, and immunity

Sleep and circadian rhythms help regulate the immune system impacting health and disease and in the context of cancer, play a role in tumor surveillance and regulation. Sleep and immunity are now thought to bi-directionally linked (20, 70). The impact of sleep and circadian rhythm on inflammatory response, leukocytes and hormones further exemplifies their potential influence with immunotherapy.

Numerous studies have shown that stimulation of the immune system by microbial challenges triggers an inflammatory response promoting sleepiness and in turn that sleep modifies the inflammatory response. Infections can increase NREM sleep through the production of cytokines such as IL-1 and TNFα (21, 71, 72). Just as sleep impacts cognitive learning and memory, it is thought that sleep also impacts the immune system’s ability to recognize learn and recall infectious stimuli modulating the adaptive immune response (20). Sleep enhancement during infection can promote host defense and increase the response to vaccination (73–76). Sleep also affects immunological memory as summarized in vaccination studies. For example, with influenza virus specific antibody titers measured 10 days after vaccination more than doubled in participants who kept their usual bedtime of 7.5 to 8 hours compared to those restricted to 4 hours of sleep. This study also showed that sleep enhances the production of Th1 effector cytokine interferonγ (77). Circadian rhythms regulate the production and function of inflammatory cytokines. For example, TNFα secretion varies in circadian fashion based on the time of an endotoxin challenge (78). Circadian rhythm and clock genes impact gene expression of key cytokines. Disruption of clock gene expression seems to be a common outcome of acute infection and it is suggested by some that the circadian clock itself is an innate immune system sensor that is disabled by infection (79).

Sleep along with circadian factors exerts a significant influence on circulating leukocyte number in the bloodstream. Studies have reported that sleep reduces the numbers of various leukocyte subsets of blood. This is thought to be due to redistribution of cells from the circulation into tissues rather than an effect on proliferation (76). Studies using sleep deprivation show an accumulation of lymphocytes in both tissue and blood. Chronic sleep deprivation which leads to the development of habitual short sleep times may cause the development of low-grade inflammation. This chronic inflammatory state is associated with an increased risk of several diseases including cancer and a decreased ability to mount an adaptive immune response (20). Clock genes and immune processes also regulate the differential maturation of leukocyte subsets as clock genes are required for the differentiation of type II lymphoid cells. Leukocyte trafficking which represents the movement of cells from the bone marrow to the bloodstream and into target organs is also under circadian control. In Bmal1 knockout mice macrophages are unable to sustain mitochondrial function, enhancing succinate dehydrogenase (SDH)-mediated mitochondrial production of reactive oxygen species as well as Hif-1α-dependent metabolic reprogramming and inflammatory damage precipitating an inflammatory and tumor- promoting cellular milieu (80). Clock genes also control neutrophil maturation (81). Furthermore specific clock genes Rev-Erbα and RORα deficient mice have negative effects on the development of and activation of dendritic cell and other antigen presenting cells (APC) critical to pathways involved with immune mediated cancer cell targeting (82). Therefore, it is increasingly clear that circadian gating is part of the core program of the immune system, and thus alteration of this regimen is likely to have widespread ramifications for disease pathogenesis (32). Furthermore, it is in slow wave sleep where high levels of growth hormone, prolactin and aldosterone and nadir levels of cortisol are present. These hormonal factors also may alter T-cell interactions. Low levels of cortisol during slow-wave sleep may allow efficient antigen-presenting cell–T-cell interactions which are important for immunomodulation and targeting cancer cells which contributes to the efficacy of cancer immune therapy (83).

## Sleep, circadian rhythms, and cancer immunotherapy

### Sleep and immunotherapy

There is a paucity of data on the relationship between sleep disturbance and immunotherapy. In an early study in lung cancer patients undergoing treatment with ICIs, Zarogoulis and associates recorded sleep disturbance using phone questionnaires and polysomnography. Interestingly for immunotherapy patients with a PD-L1 expression greater than 50%, disease response was rapid and associated sleep disturbances decreased rapidly. In contrast, in patients on standard chemotherapy, those with both partial response and stable disease continued to have sleep disturbances. They concluded that although ICIs did not induce sleep disturbance, treatment response may improve sleep disturbances (84). Another study by the same group showed that upon diagnosis, lung cancer patients had sleep disturbances including early morning awakenings, late sleep onset, prolonged nocturnal waking periods, daytime sleepiness, and unrefreshed sleep. These symptoms improved quickly in patients with a PDL-1 expression greater than 80% during the first 4 months of treatment due to the rapid response of the tumor to immunotherapy. There was no difference in symptom control seen between patients who received nivolumab or pembrolizumab (85).

Recent studies have demonstrated that ICIs can impact symptoms which cluster with sleep disruption such as cancer-related fatigue (CRF). These symptoms may present coincidentally as cancer-related symptoms clusters sharing common inflammatory, hormonal, autonomic nervous system, and hypothalamic-pituitary axis abnormalities. Hajjar and colleagues found that in 88 patients with advanced metastatic cancers undergoing immunotherapy, fatigue was identified in 66%. High level fatigue was found in 34%. This study is among the first to describe the microbiome in patients on immunotherapy which also has an important immune mediated impact, potentially affecting ICI effectiveness and irAEs. The study was able to show that there was a correlation of *Eubacterium hallii* that was negatively associated with fatigue severity scores whereas those patients with *Cosenzaea* sp. had higher fatigue scores (86). In a meta-analysis in subjects on ICI therapy, CTLA-4 inhibitors are associated with a higher risk of all and high-grade fatigue compared with control regimens, whereas PD-1 inhibitors are associated with a lower risk of all- and high-grade fatigue compared with control regimens. Although ICIs have revolutionized the treatment of certain cancers, many patients do not respond to ICIs and treatment outcomes vary disproportionately between cancer types (87). Therefore there is a significant interest how lifestyles, diet, and psychosocial factors including sleep quality determine the outcomes and ICI management (88). The impact of ICI therapy on sleep and related symptoms and the association with microbiome composition present opportunities to potentially augment the efficacy and tolerance of immunotherapy.

### Circadian rhythm and immunotherapy

The human immune system is equipped to keep unnatural cell growth in check and aide in cancer suppression (69). Given the circadian clock’s influence on immune recognition and elimination of cancer cells, it is unsurprising that there is evolving evidence for the connection between circadian rhythms and cancer immunotherapy. In a metastatic melanoma murine model, BMAL1 is responsible for T-cell activation and the expression of CTLA-4, PD-1 and PD-L1 (89). Enrichment of clock gene pathways in animal studies increase PD-L1 expression and enhance T cell receptor signaling (90). In normal lung tissue, clock genes Per1 and Cry2 have been linked to the expression of CD4+ T cells and PD-1 expression follows a circadian rhythm (91). In clock gene RORγ knockout mice, the presence of PD-1 Type 17 cells and levels of PD-1 is decreased (92).

The circadian clock also affects the functional response of CD8 T-cells to antigen presentation by dendritic cells, the core aspect of the immune response against pathogens and cancer cells. This early T-cell receptor response was shown to impact T-cell receptor signaling such as activation, proliferation factor functions. The influence of circadian rhythms on the functional aspect of T-cell development, response to antigens, and trafficking is well recognized (69). Although there is no direct evidence that eloquently describes interplay between circadian rhythms and immunotherapy response, it is an exciting area of study and has the potential to transform immunotherapy (88).

Circadian rhythms are being considered for the prevention and management diseases including cancer (60). There are 3 broad approaches using our knowledge about circadian rhythms to impact cancer outcomes. First, lifestyle management, including sleep hygiene, diet composition and timing, and exercise timing, may be used to entrain a robust circadian rhythm. Consistent daily behavior patterns and sleep and eating may significantly reduce the risk of cancer (88). Studies in mice, utilizing rhythmic food intake have shown that these signals driven contribute to driving rhythms in liver gene expression and metabolic functions outweighing the influence of even the cell-autonomous hepatic clock (93). In addition, microbiome modification by lifestyle changes is an area of active study in patients on immunotherapy, as recently reviewed by Wargo and associates (94). Secondly, chronotherapy, or the timing of medication administration, can be used to target tumors while preventing adverse off-target effects. Nelson and Levi have shown that the time of day of infusion of both PD-1 and PD-L1 inhibitors showed clinically significant association with increased survival in patients which a variety of cancer types (95, 96). Finally, recent advancements on how to enhance our circadian clock through pharmacological targeting of circadian clock components that are already providing new preventive and therapeutic strategies for several diseases, including metabolic syndrome and cancer (97). A new class of drugs targeting circadian clock genes (“clock drugs”) act through targeting components of the clock circadian clock and have shown early promise in modulating immunotherapy (98). Clock drugs directly target the circadian clock components including, RORγ, REV–ERB’s (99). Furthermore, specific clock genes REV-ERBα and RORα deficient mice have negative effects on the development of and activation of dendritic cell and other antigen presenting cells critical to pathways involved with immune-mediated cancer cell targeting (82). In a series of experiments done by Hu and colleagues, RORγ agonists can act as monotherapy *in vivo* and display anti-tumor properties, including boosting the activity of TH17 cells. When treated with RORγ agonists, T-cells are more resistant to PD-L1 inhibition which is critical in suppressing anti-tumor activity. Supplementation of a RORγ agonist during ex-vivo expansion during chimeric antigen receptor (CAR)-T cell engineering, increased the antitumor activity of TH17 cells. The function of the CAR Type 17 cells is elevated when re-exposed to tumor with increase cytokine production including IL-17A and IFNγ. Moreover, mice with RORγt-primed T cells are protected after cancer cell inoculation (92, 100).

Circadian rhythms may also indicate treatment prognosis in immunotherapy with a recent study finding that in patients undergoing immunotherapy for lung adenocarcinoma, a circadian rhythm gene related signal could serve as an independent indicator for prognosis. This circadian rhythm genetic marker was found to be upregulated in cancer samples (101). These data taken together, suggest that circadian rhythms are likely to be harnessed in the future to augment the impact of immunotherapy in the treatment of cancer.

## Future directions

As the role of ICIs expand, the impact of sleep, circadian rhythms, immunity, and immunotherapy requires further study. There are several unanswered and significant questions.

■ Are sleep and circadian rhythms a biomarker for prognosis in patients on immunotherapy as they have been shown in other therapies?
■ What are the interactions between sleep and circadian and other modifiable lifestyle factors such as diet?
■ Do these factors impact the microbiome and thereby affect treatment related outcomes with immunotherapy?
■ How will chronotherapy best be utilized to boost on target effects of immunotherapy?
■ Can we exploit the interconnection between the circadian clock genes and the immune system to drive more effective and safer immunotherapy approaches, minimizing auto-immunity?

It is the answers to these and other intriguing questions that lie the heart reaping of the promise of immunotherapy, that further exploration of these complex relationship hold.

## Conclusion

Immunotherapy has transformed cancer treatment, and given its impact on the immune system, the role of ICI as it relates to sleep and circadian rhythm continue to unfold. Clearly an intricate complex relationship exists in the microenvironment with immunity, sleep and the tumor, and these may further vary based on the cancer, addition of standard chemotherapy, pre-existing patient factors and irAEs. Sleep and circadian rhythms may offer tools to better utilize immunotherapy in the care of cancer patients, leading to better treatment outcome, reduced symptom burden, and increased quality of life.

## Author contributions

DB: Conceptualization, Formal analysis, Methodology, Project administration, Resources, Supervision, Validation, Visualization, Writing – original draft, Writing – review and editing. LB: Conceptualization, Writing – review and editing. AS: Conceptualization, Writing – review and editing. EM: Conceptualization, Writing – review and editing. SF: Conceptualization, Visualization, Writing – review and editing.
